# Structural Cerebellar Abnormalities and Parkinsonism in Patients with 22q11.2 Deletion Syndrome

**DOI:** 10.3390/brainsci12111533

**Published:** 2022-11-12

**Authors:** Claudia Piervincenzi, Martina Fanella, Nikolaos Petsas, Marianna Frascarelli, Alessandra Morano, Tommaso Accinni, Fabio Di Fabio, Carlo Di Bonaventura, Alfredo Berardelli, Patrizia Pantano

**Affiliations:** 1 Department of Human Neurosciences, Sapienza University of Rome, 00185 Rome, Italy; 2 Department of Neurology, Fabrizio Spaziani Hospital, 03100 Frosinone, Italy; 3 IRCCS NEUROMED, 86077 Pozzilli, Italy

**Keywords:** 22q11.2 deletion syndrome, parkinsonism, cerebellum, voxel-based morphometry (VBM)

## Abstract

**Background:** The phenotypic expression of 22q11.2 deletion syndrome (22q11.2DS) is variable and may include cognitive, psychiatric, and neurological manifestations, e.g., parkinsonism. We investigated brain structural alterations in patients with 22q11.2DS with and without parkinsonism (Park+ and Park−) in comparison with healthy controls (HCs). **Methods**: Voxel-based morphometry was performed on 3D T1-weighted MR images to explore gray matter volume (GMV) differences between 29 patients (15 Park+, 14 Park−), selected from a consecutive series of 56 adults diagnosed with 22q11.2DS, and 24 HCs. One-way ANOVA and multiple linear regression analyses were performed to explore group differences in GMV and correlations between clinical scores (MDS-UPDR-III and MoCA scores) and structural alterations. **Results**: Significant between-group differences in GMV were found in the cerebellum, specifically in bilateral lobes VIII and left Crus II, as well as in the left superior occipital gyrus. Although both Park+ and Park− patients showed GMV decrements in these regions with respect to HCs, GMV loss in the right lobe VIII and left Crus II was greater in Park+ than in Park− patients. GMV loss did not correlate with clinical scores. **Conclusions**: Patients with 22q11.2DS and parkinsonism manifest specific cerebellar volume alterations, supporting the hypothesis of neurodegenerative processes in specific cerebellar regions as a putative pathophysiological mechanism responsible for parkinsonism in patients with 22q11.2DS.

## 1. Introduction

22q11.2 deletion syndrome (22q11.2DS), also known as DiGeorge or velocardiofacial syndrome, is a multisystem disorder caused by a chromosomal microdeletion involving a 3 Mb segment on the long arm of chromosome 22. It is the most common microdeletion in humans, affecting one in every 4000 individuals [1,2,3]. The phenotypic expression of 22q11.2DS is highly variable [4] and may include cognitive, psychiatric, and neurological manifestations.

Most patients have a borderline intellectual level, whereas severe intellectual disabilities are observed less frequently [5,6]. The neurocognitive domains showing the greatest impairment are attention, working memory, visual–spatial, and executive functions [7,8]. Left-handedness has recently emerged as a common feature in these patients [9]. The most frequent psychiatric manifestations are schizophrenia [10,11], autism spectrum disorder [12], attention-deficit/hyperactivity disorder [13], anxiety, and depression [14,15].

The syndrome is also associated with neurological disorders like epilepsy and movement disorders, such as parkinsonism [9,16]. In 22q11.2DS patients, parkinsonism is characterized by asymmetric clinical signs and a positive response to L-dopa and dopamine agonist treatment. 

Although the association between 22q11.2DS and parkinsonism was first observed in 1998 [17], only a few case reports and case series have been published that describe the early onset of symptoms in subjects with neither a family history of parkinsonism nor gene mutations commonly associated with early-onset Parkinson’s disease (PD) [18,19,20,21,22,23,24]. A recent cross-sectional study revealed that among neurological manifestations of 22q11.2DS, parkinsonism was present in 55% of patients [9], confirming it as a common feature of the syndrome. Although some neuropathological studies reported a loss of midbrain dopaminergic neurons and variable Lewy body neuropathology in 22q11.2DS patients with parkinsonism [19,20,22], there is still no neuroimaging evidence of potential relevance in terms of parkinsonism and PD in individuals with 22q11.2DS.

### Aims and Objectives

The present study aimed to identify magnetic resonance imaging (MRI)-related biomarkers of parkinsonism in patients with 22q11.2DS by examining possible whole-brain structural alterations in 22q11.2DS patients with (Park+) and without parkinsonism (Park−). We hypothesized that the presence of parkinsonism in individuals with 22q11.2DS could be associated with specific gray matter loss in motor-related supra- and/or infratentorial brain regions.

## 2. Methods

### 2.1. Study Design and Participants 

All patients belonged to a previously published study focused on the neurological manifestations of fifty-six adults diagnosed with 22q11.2DS (for details see [9]). Patients were recruited at the Department of Human Neurosciences, Sapienza University of Rome, Policlinico Umberto I Hospital, Italy. These subjects came from different Italian regions and were members of AIDEL22 (Associazione Italiana Delezione 22). The diagnosis was genetically confirmed by means of fluorescence in situ hybridization or array-comparative genomic hybridization. Twenty-nine of these patients (21 males, mean age ± standard deviation (SD): 26.6 ± 8.1 years) underwent MRI scanning and were included in the present study. The study cohort included 15 Park+ (11 males, mean age ± SD: 29.5 ± 9.8 years) and 14 Park− patients (10 males, mean age ± SD: 23.5 ± 4.1 years). Seven Park+ and five Park− patients were taking medications with potential drug-induced parkinsonism effects, i.e., atypical antipsychotics, at the time the study was performed. Twenty-four healthy controls (9 males, mean age ± SD: 29.8 ± 9.1 years) were also enrolled for comparison.

### 2.2. Clinical and Neuropsychological Assessment

All patients underwent a thorough neurological examination. Dominance in manual skills was established using the Edinburgh Handedness Inventory [25]. Parkinsonism was diagnosed according to the most recent clinical criteria [26] and was rated using the Movement Disorder Society-sponsored revision of the Unified Parkinson’s Disease Rating Scale part III (MDS-UPDRS-III) [27], the most widely used clinical rating scale to evaluate motor symptoms in PD [28]. In our cohort, parkinsonism was mainly characterized by the presence of bradykinesia, rigidity, and parkinsonian gait, whereas tremor was less frequently present. Cognitive performance was assessed using the Montreal Cognitive Assessment (MoCA) [29]. MoCA is a brief, simple, and reliable screening tool for the assessment of cognitive impairment. It checks language, memory, visuo–spatial thinking, reasoning, and orientation skills. The total score ranges from 0 to 30 points, and a total score of 26 and higher is considered normal [29].

### 2.3. MRI Data Acquisition and Voxel-Based Morphometry (VBM)

MRI scans were acquired using a 3-Tesla scanner (Siemens Magnetom Verio) with a 12-channel head coil designed for parallel imaging (GRAPPA). Whole-brain T1-weighted magnetic-prepared rapid-gradient echo (MPRAGE) sequence images were acquired for each subject, with the following parameters: repetition time (TR) = 2400 ms, echo time (TE) = 2.12 ms, inversion time (TI) = 1000 ms, flip angle = 8°, field of view (FOV) = 256 mm, matrix = 256 × 256, 176 sagittal slices 1-mm thick, no gap.

The VBM analysis was conducted using the Computational Anatomy Toolbox (CAT12, accessed on 1 April 2022, http://dbm.neuro.uni-jena.de/cat/), an extension toolbox of Statistical Parametric Mapping software (SPM12, accessed on 1 April 2022, http://www.fil.ion.ucl.ac.uk/spm/software/spm12). The VBM analysis was performed using the default settings described in the CAT12 manual (accessed on 1 April 2022, http://dbm.neuro.uni-jena.de/cat12/CAT12-Manual.pdf). Briefly, T1-weighted images were spatially normalized and segmented into gray matter (GM), white matter (WM), and cerebrospinal fluid tissue classes according to the DARTEL approach with default settings in 1.5-mm cubic resolution and Montreal Neurological Institute (MNI) space. The normalized maps were modulated with the resulting Jacobian determinant maps to preserve GM volume (GMV) of native space and smoothed using a Gaussian filter (8-mm full-width half-maximum). The total intracranial volume (TIV) of each subject was calculated and used as a covariate for further statistical analyses. 

### 2.4. Statistical Analyses

Statistical analyses of demographic, clinical, and neuropsychological parameters were performed using SPSS statistics software (version 22.0). Between-group differences (all patients vs. controls and Park+ vs. Park− patients) were tested using the Mann–Whitney U test and Fisher’s exact test for continuous and dichotomous variables, respectively (*p* < 0.05 for null hypothesis rejection). 

The generalized linear model implemented in SPM12 was used to assess potential between-group differences in GMV. Individual smoothed GM maps were entered into a one-way analysis of variance design, including age, gender, handedness, and TIV as nuisance covariates to control for these variables within the study population. After checking for a significant main effect of group, a mask was created at *p* < 0.05, and family-wise error (FWE)-corrected (cluster size ≥ 20 voxels), including the average effect of condition. This mask was derived from thresholding the F-statistic image and was used to identify significant clusters of altered GMV in post-hoc between-group comparisons by constraining the analysis to areas within the mask. Since several patients took medications with potential drug-induced parkinsonism effects, i.e., atypical antipsychotics (7 Park+ and 5 Park− patients), a subgroup VBM analysis was performed by removing those patients (see Methods section in Supplementary Materials).

For Park+ and Park− patients, multiple regression analyses were performed to investigate possible correlations between MDS-UPDRS-III (only in Park+) and MoCA scores and GMV alterations. Multiple regression analyses were restricted to voxels displaying significant GMV alterations by applying the above-mentioned mask. Anatomical localization of significant clusters was performed using the automated anatomical labeling (AAL) toolbox of SPM [30].

## 3. Results

### 3.1. Clinical Findings

Descriptive statistics for demographic and clinical features in Park+ and Park− patients and controls are reported in Table 1. There were no significant differences in age between patients and controls or between Park+ and Park− patients, whereas patients and controls significantly differed in sex distribution (*p* = 0.014) and left-handedness (*p* = 0.027). There were no significant differences in clinical/neuropsychological measures between Park+ and Park− patients. Among Park+ patients, the mean MDS-UPDRS-III score was 9.9 (SD 8.6).

### 3.2. VBM

**Figure 1:**
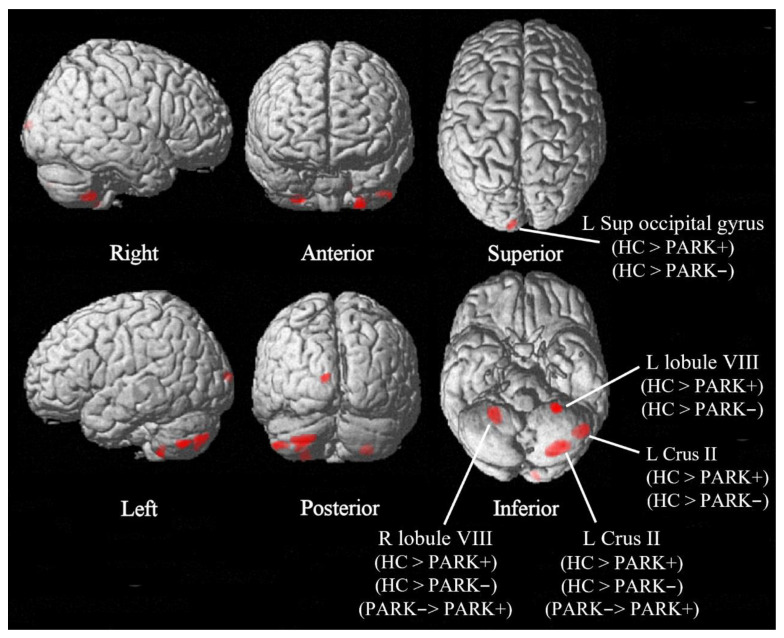
Voxel-based morphometry (VBM) F-test results comparing healthy controls (HCs) and 22q11.2DS patients with and without parkinsonism (Park+ and Park−). Significant group differences (*p* < 0.05, family-wise error-corrected, k ≥ 20 voxels) are shown in red on a 3D render of the standard brain (six different views of the render are shown). Post-hoc analysis results are also reported for each cluster of the F-statistic image.

Average effect of condition F test showed significant (*p* < 0.05, FWE-corrected) clusters of GMV alterations, mainly located in the cerebellum, specifically in bilateral cerebellar lobes VIII and left Crus II, as well as in the left superior occipital gyrus. Post-hoc analyses showed that both Park+ and Park− patients showed decrements in GMV in these regions with respect to controls. However, Park+ patients also showed lower GMV in the cerebellum, specifically in the right cerebellar lobe VIII and left Crus II, with respect to Park− patients (Figure 1, Table 2). These results were confirmed by the subgroup analysis performed on patients who were not taking antipsychotics (see Results section, Supplementary Figure S1 and Supplementary Table S1 in Supplementary Materials).

### 3.3. Correlation Analyses

No significant correlations were found between MDS-UPDRS-III score and GMV reductions in Park+ patients or between MoCA score and GMV changes in either patient group.

## 4. Discussion

In the present study, we found that patients with 22q11.2DS showed prominent reductions in GMV in the cerebellum as compared with healthy controls, and that structural alterations in specific cerebellar lobes, i.e., lobules VIII and Crus II, differentiated 22q11.2DS patients with parkinsonism from those without motor symptoms. The cerebellum is known to influence motor and cognitive operations. The anterior lobe and lobules VI and VIII are predominantly sensorimotor, while the posterior lobe (including Crus II) contributes to higher level processes such as language, spatial, executive, working memory, and affective tasks [31]. Our neuroimaging findings provide further insight into the pathophysiological mechanisms underpinning parkinsonism in 22q11.2DS.

### 4.1. GMV Alterations in Park+ and Park− Patients

Both Park+ and Park− 22q11.2DS patients showed major GMV reductions in the cerebellum with respect to healthy controls. The cerebellum seems to be a critical but understudied component of the 22q11DS neuro-endophenotype; although previous works showed global cerebellar atrophy in individuals with 22q11.2DS [32,33,34], only a few studies have investigated regional cerebellar substructure in 22q11DS. Two studies focused solely on midsagittal regions of interest, reporting a significant reduction in vermian areas in individuals with 22q11DS [35,36]. In a more recent study, Schmitt and colleagues [37] performed a systematic survey of regional cerebellar volumes in 22q11DS patients using a large sample of patients (N = 79) and found that individuals with 22q11DS had, on average, smaller total cerebellar volumes relative to typically developing subjects. The largest differences were found in Crus I, Crus II, lobule VIIB, and lobule VIIIA. Our results are in agreement with the findings of Schmitt and colleagues and confirm that lobules of the posterior cerebellum are affected in individuals with 22q11DS.

### 4.2. Differences in GMV Alterations between Park+ and Park− Patients

Although cerebellar atrophy has previously been reported in 22q11.2DS patients, no study investigated the possible role of cerebellar involvement in parkinsonian signs. A relevant finding of this study is greater volume loss in specific cerebellar regions, i.e., right lobule VIII and left Crus II, in Park+ patients as compared with controls and Park− patients. 

Along with the anterior cerebellum, cerebellar lobule VIII has been identified as a motor cerebellar lobule by several task-based and resting-state functional MRI studies [38,39,40], while cerebellar Crus II is generally thought to be crucial for cognition representation [41,42]. Interestingly, Crus II seems to play an important role in motor imagery [39]. Furthermore, specific engagement of the left lateral cerebellum in visual processing of body motion has been suggested [43]. Cerebellar Crus II is also involved in motor timing [44] and, along with the anterior cerebellum, in performing complex actions [45]. 

Recent evidence has highlighted the role of the cerebellum in PD. The cerebellum has a strong projection to the striatum by way of the thalamus and may influence the pathways involved in basal ganglia processing through an integrated functional network. The discovery of these reciprocal connections between the basal ganglia and cerebellum provides an anatomical basis to explain the role of the cerebellum in PD [46,47]. Studies that have investigated the cerebellum have demonstrated that PD patients have cerebellar atrophy [48,49]. Moreover, some authors have suggested the presence of cerebellar functional changes during the progression of PD. In a relatively early stage, the cerebellum could have a compensatory effect in order to maintain relatively normal motor function, but this compensatory effect apparently diminishes throughout disease progression and fails at advanced stages [50,51].

In accordance with these data, we might speculate that in 22q11.2DS patients, atrophy of strategic motor cerebellar lobules could determine functional changes in cerebellar-striatal networks, resulting in parkinsonism despite the absence of anatomical changes in the basal ganglia.

Lastly, the present results were confirmed by the subgroup analysis performed on patients who were not taking neuroleptic drugs. This interesting finding might suggest that the role of antipsychotics is not causative and that these drugs can only facilitate the emergence of parkinsonian signs in subjects with genetic susceptibility.

### 4.3. Study Strengths and Limitations

Our study has some strengths and limitations. First, we focused for the first time on parkinsonism, a common but understudied feature of this rare genetic condition [9,16]. Second, we used a well-established image analysis technique (VBM) to compare whole-brain GMV differences between groups. The effects of age, gender, handedness, and total intracranial volume were controlled statistically in the VBM analysis, to ensure that GMV differences were not due to possible differences in these parameters. Third, we addressed the confounding role of neuroleptics (drug-induced parkinsonism) by demonstrating that GMV differences between Park+ and Park− patients are still present in the subgroup of patients who were not taking atypical antipsychotics.

The main limitation of the present work is the relatively small number of patients enrolled. The small number of patients with parkinsonism (N = 15) may also explain the lack of significant correlations between clinical scales (MDS-UPDRS and MoCA scores) and GMV alterations. Further research on larger samples will be critical to better characterize the effect of aberrant cerebellar morphology in individuals with 22q11DS and parkinsonism. Another limitation is the lack of a comprehensive neuropsychological evaluation. Despite the absence of a significant difference in MoCA scores between Park+ and Park− patients seeming to suggest that the present findings are not attributable to differences in cognitive level, this finding must be confirmed in future studies including extensive neuropsychological assessment.

## 5. Conclusions

Our study provides further evidence that cerebellar gray matter volume reduction is a common feature in 22q11.2DS patients and demonstrates that patients with parkinsonism are characterized by a higher atrophy rate of strategic motor cerebellar lobules. The present findings support the hypothesis that neurodegenerative processes in specific brain regions and indirect involvement of cerebellar-striatal networks could play a role in the pathophysiology of parkinsonism in 22q11.2DS patients. The results of the present study suggest further investigation into the functionality of cerebellar processing in such patients. By using advanced MRI sequences (i.e., functional MRI and diffusion-weighted imaging) to explore structural/functional connectivity abnormalities in the cerebellar-striatal networks, future studies may shed light on the role of cerebellar damage in individuals with 22q11.2DS and parkinsonism.

## Figures and Tables

**Table 1:** Demographic and clinical characteristics of healthy controls (HCs) and patients with 22q11.2 deletion syndrome (22q11DS) with and without parkinsonism (Park+ and Park−). Values are reported as the mean ± standard deviation; n, number; y, years; ns, not statistically significant.

	HCs (N = 24)	22q11.2DS (N = 29)	P *	Park+ (N = 15)	Park− (N = 14)	P *
**Demographic/** **clinical features**						
Age	29.8 ± 9.1	26.6 ± 8.1	ns	29.5 ± 9.8	23.5 ± 4.1	ns
Female/male, n	15/9	8/21	0.014	4/11	4/10	ns
Left-handedness (yes/no)	0/20	6/23	0.027	3/12	3/11	ns
Neuroleptics (yes/no)	-	12/17	-	7/8	5/9	ns
**Neuropsychological scores**						
MDS-UPDRS-III	-	9.9 ± 8.6 §	-	9.9 ± 8.6	-	-
MoCA	-	23.0 ± 4.0	-	22.3 ± 5.1	23.9 ± 1.8	ns

MDS-UPDRS-III: Movement Disorder Society-sponsored revision of the Unified Parkinson’s Disease Rating Scale, part III; MoCA: Montreal Cognitive Assessment. § in Park+ 22q11.2DS patients. P * Mann–Whitney U-test and Fisher’s exact test for continuous and dichotomous variables, respectively (*p* < 0.05).

**Table 2:** Voxel-based morphometry (VBM) results comparing healthy controls (HCs) and 22q11.2DS patients with and without parkinsonism (Park+ and Park−). Anatomical localization of significant (*p* < 0.05 family-wise error-corrected, k ≥ 20 voxels) clusters was performed using the automated anatomical labeling toolbox of Statistical Parametric Mapping. L = left, R = right.

K	*F*	*P*	MNI Coordinates (mm)			Brain region	*Post hoc*
			x	y	Z		
252	36.23	<0.001	28	−48	−50	R Cerebellum, lobule VIII	HCs > Park+ HCs > Park− Park− > Park+
172	32.83	<0.001	−24	−42	−58	L Cerebellum, lobule VIII	HCs > Park+ HCs > Park−
435	26.70	<0.001	−28	−76	−44	L Cerebellum, Crus II	HCs > Park+ HCs > Park− Park− > Park+
78	24.54	0.001	−8	−99	9	L Superior occipital gyrus	HCs > Park+ HCs > Park−
195	24.53	<0.001	−48	−62	−48	L Cerebellum, Crus II	HCs > Park+ HCs > Park−

## Data Availability

The datasets presented in this article are not readily available because of patient confidentiality and participant privacy restrictions. Requests to access the datasets should be directed to the corresponding author.

## Supplementary Materials

The following supporting information can be downloaded at: https://www.mdpi.com/article/10.3390/brainsci12111533/s1, Figure S1: Voxel-based morphometry (VBM) F-test result comparing healthy controls (HC) and 22q11.2DS patients with and without parkinsonism (Park+ and Park−); Table S1: Voxel-based morphometry (VBM) result comparing healthy controls and 22q11.2DS patients with and without parkinsonism (Park+ and Park−). Anatomical localization of significant (*p* < 0.05 FDR corrected, k ≥ 20 voxels) clusters was performed using the AAL toolbox of SPM.
