# Supplementary material for: Structural Cerebellar Abnormalities and Parkinsonism in Patients with 22q11.2 Deletion Syndrome
Source: Brain Sci. 2022 Nov 12;12(11):1533. doi: 10.3390/brainsci12111533 (PMC9688398; doi:10.3390/brainsci12111533)
Supplement: Supplementary file 1 [file brainsci-12-01533-s001.zip › brainsci-2026053-supplementary.pdf]

Supplementary Materials.

Structural cerebellar abnormalities and parkinsonism in patients with 22q11.2 deletion syndrome

Methods

A subgroup VBM analysis was performed including only those patients which did not take medication with potential drug-induced parkinsonism effects (8 Park+ and 9 Park−). Individual smoothed GM volume maps were entered into an one-way ANOVA design, including age, gender, handedness and TIV as nuisance covariates. Due to the exploratory nature of this analysis and the small sample size of patient subgroups, results were corrected using False Discovery Rate (FDR) correction [1] for multiple-comparisons ($p < 0.05$, cluster size ≥20 voxels).

Results

Average effect of condition F test showed significant clusters of GM alterations located in bilateral cerebellar lobes VIII and left Crus II, and in the anterior cingulate cortex. Post-hoc analyses showed that both Park+ and Park− showed decrements in GM volume in these regions respect to controls; Park+ also showed lower GM volumes in the right cerebellar lobe VIII and left Crus II respect to Park− (Supplementary Figure S1, Supplementary Table S1).

Supplementary Table S1. Voxel-based morphometry (VBM) result comparing healthy controls and 22q11.2DS patients with and without parkinsonism (Park+ and Park−). Anatomical localization of significant ($p<0.05$ FDR corrected, $k \geq 20$ voxels) clusters was performed using the AAL toolbox of SPM. L=left, R=right

k	*F*	*P*	MNI coordinates (mm)			Brain region	*Post hoc*
			x	y	z		
395	19.39	0.042	27	−48	−51	R Cerebellum, lobule VIII	HC > Park+
	18.95	0.042	16	−68	−51	R Cerebellum, lobule VIII	HC > Park−<br>Park− > Park+
50	18.71	0.042	−26	−42	−58	L Cerebellum, lobule VIII	HC > Park+<br>HC > Park−
76	15.34	0.046	−21	−81	−42	L Cerebellum, Crus II	HC > Park+<br>HC > Park−<br>Park− > Park+
29	14.90	0.047	40	−54	−60	R Cerebellum, lobule VIII	HC > Park+<br>HC > Park−
25	14.69	0.047	15	36	21	R Anterior cingulate cortex	HC > Park+<br>HC > Park−

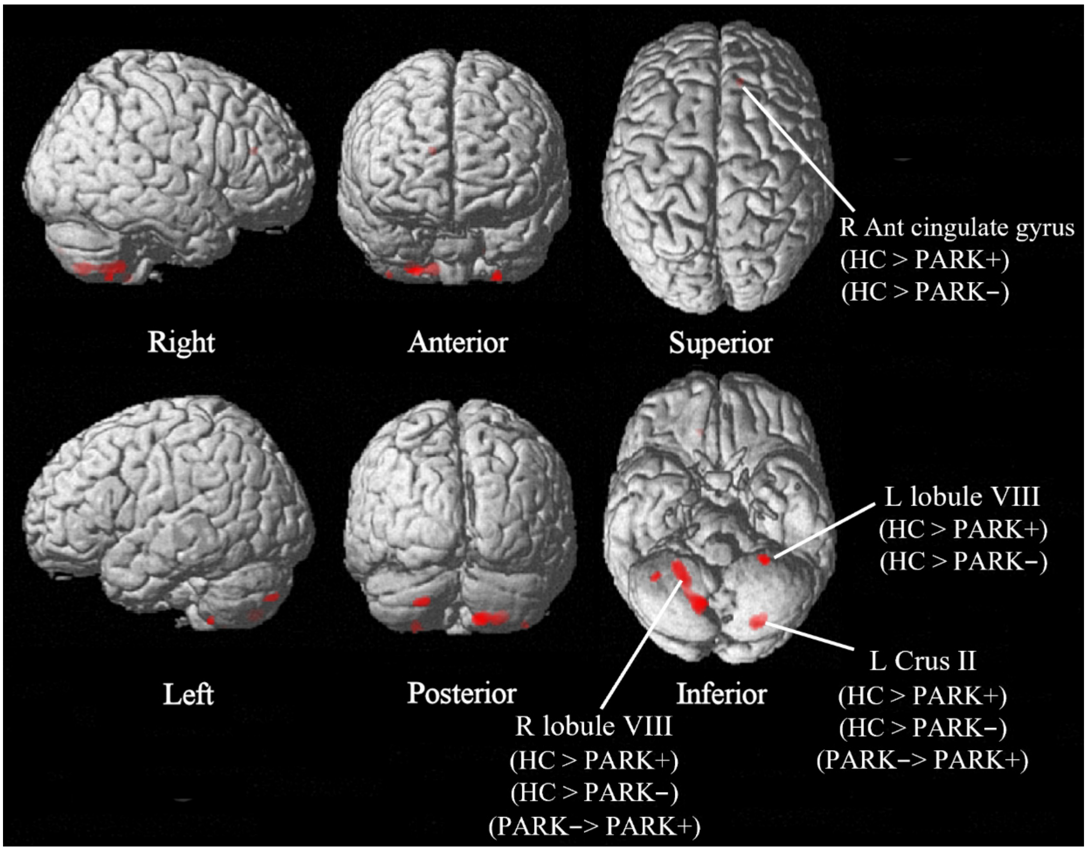

Supplementary Figure S1. Voxel-based morphometry (VBM) F-test result comparing healthy controls (HC) and 22q11.2DS patients with and without parkinsonism (Park+ and Park−). Significant group differences ($p < 0.05$, FDR corrected, $k \geq 20$ voxels) are shown in red on a 3D render of the standard brain (six different views of the render are shown). Post-hoc analysis results are also reported for each cluster of the F-statistic image.

References

1. Benjamini Y, Hochberg Y. Controlling the False Discovery Rate: A Practical and Powerful Approach to Multiple Testing. *Journal of the Royal Statistical Society: Series B (Methodological)*,1995, 57, 289–300. https://doi.org/10.1111/j.2517-6161.1995.tb02031.x
